# Assessment of emphysema with simulated low-dose dark-field radiography

**DOI:** 10.1186/s41747-026-00796-x

**Published:** 2026-09-10

**Authors:** Henriette Bast, Rafael C. Schick, Thomas Koehler, Florian T. Gassert, Alexander W. Marka, Andreas P. Sauter, Bernhard Renger, Gregor Zimmermann, Marcus R. Makowski, Franz Pfeiffer, Daniela Pfeiffer

**Affiliations:** 1 Chair of Biomedical Physics, Department of Physics, TUM School of Natural Sciences, Technical University of Munich, Garching, Germany; 2 Munich Institute of Biomedical Engineering, Technical University of Munich, Garching, Germany; 3 Institute for Diagnostic and Interventional Radiology, School of Medicine and Health, TUM Klinikum, Technical University of Munich (TUM), Munich, Germany; 4 Philips Innovative Technologies, Hamburg, Germany; 5 Munich Institute for Advanced Study, Technical University of Munich, Garching, Germany; 6 Division of Respiratory Medicine, Department of Internal Medicine I, School of Medicine and Health, TUM Klinikum, Technical University of Munich (TUM), Munich, Germany

**Keywords:** Pulmonary emphysema, Radiation dosage, Radiography, x-ray

## Abstract

**Objective:**

Dark-field radiography allows the visualization of lung tissue changes associated with pulmonary emphysema. Simulations were used to investigate the impact of radiation dose reduction on the diagnostic accuracy of emphysema detection.

**Materials and methods:**

Dark-field and attenuation chest radiographs acquired during an ongoing clinical study were used to simulate multiple low-dose counterparts corresponding to median effective doses between 7 and 18 µSv. All study participants had undergone a medically indicated chest computed tomography scan that showed either pulmonary emphysema or no lung pathologies. In a randomized reader study, three radiologists independently assessed the simulated low-dose dark-field and attenuation radiographs as well as the original images for the presence of emphysema. The area under the curves (AUC) of the receiver operating characteristics (ROC) were analyzed using Obuchowski’s method. ROC curves were also generated by using different values of the dark-field coefficient to differentiate between absent and at least mild emphysema.

**Results:**

The original (full-dose) images and simulated low-dose images of 93 participants (57 males, 36 females) were included, with participants’ median age of 66 years (interquartile range 54 to 73 years). The radiologists’ ROC curves of the simulated low-dose images at 11 µSv (AUC = 0.84) did not differ significantly (*p *= 0.177) from the full-dose curve (AUC = 0.86). For all dose levels, the dark-field coefficient could distinguish between absent and at least mild emphysema with an AUC of 0.85.

**Conclusion:**

Radiation dose reduction in dark-field imaging for emphysema assessment might be feasible, with median effective doses potentially as low as 11 µSv.

**Key Points:**

- ***Question*** How does radiation dose reduction affect the diagnostic potential of dark-field chest radiographs?
- ***Findings*** For actual, regular-dose (36 µSv) dark-field radiographs, AUC was 0.86, and 0.84 for simulated low-dose images. Down to 7 µSv, the dark-field coefficient AUC was 0.85.
- ***Relevance statement*** Simulations suggest that the radiation dose in dark-field chest imaging could be lowered to 11 µSv, indicating the technique’s potential for ultra-low-dose imaging, which could be especially beneficial for potential clinical screening applications in the future.

**Graphical Abstract:**

**Figure:**
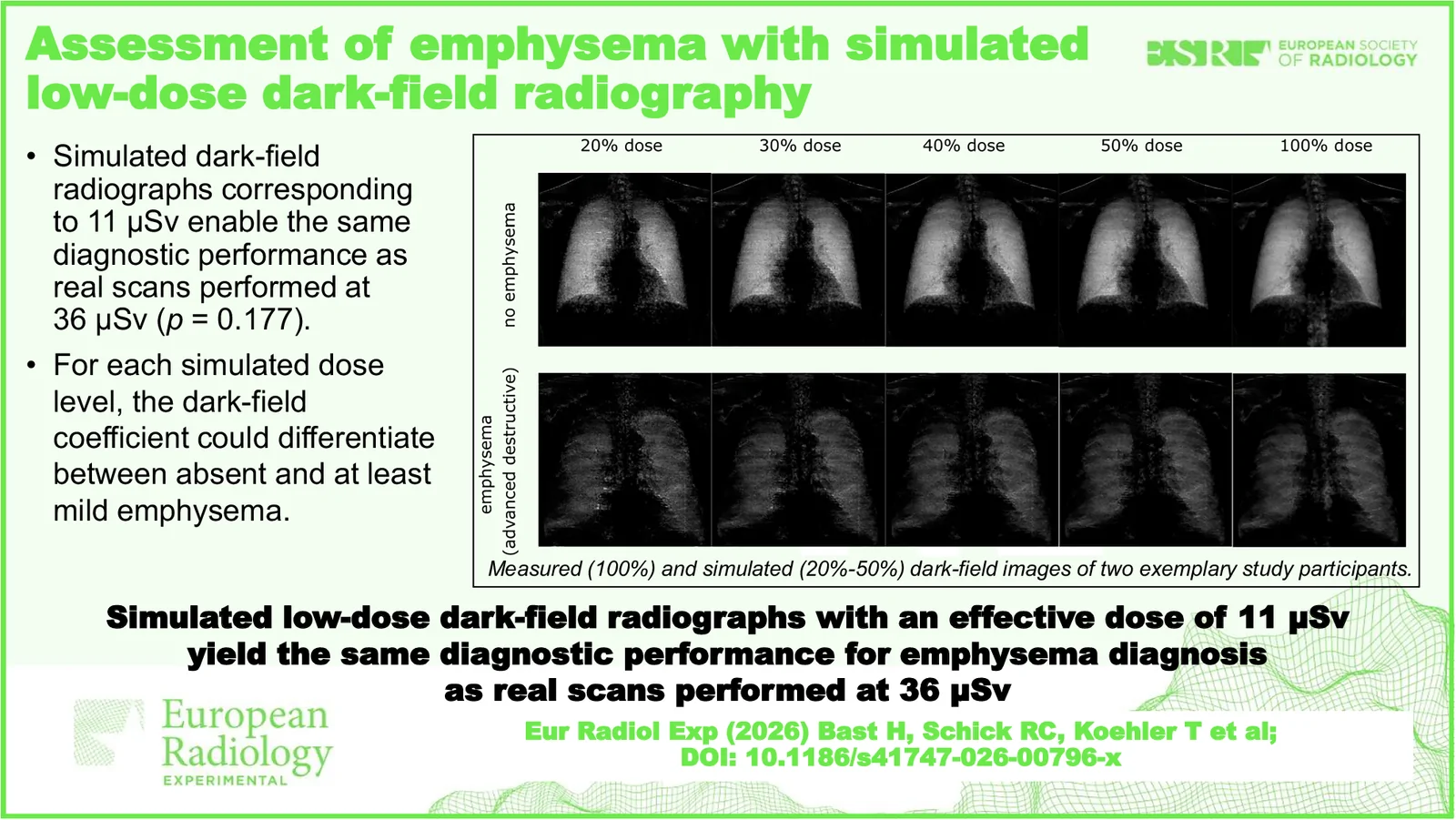

## Background

First clinical studies on dark-field radiographs of human lungs showed that the lungs’ dark-field signal is affected by various lung diseases [1–7]. While healthy lungs exhibit a strong and homogeneous dark-field signal [2], emphysema and COVID-19 pneumonia lead to a reduced and patchy dark-field image [1, 3, 6, 8], and a pneumothorax causes total signal loss [9]. Quantitatively, the so-called dark-field coefficient has been introduced to measure such changes [2]. Regarding the diagnosis and staging of pulmonary emphysema, dark-field chest radiography outperforms conventional chest radiography [10]. Hence, dark-field radiography is a promising new imaging technique, particularly suitable for the assessment of emphysema in patients with chronic obstructive pulmonary disease (COPD).

These published clinical studies were performed using a grating-based dark-field prototype system located at the University Hospital Rechts der Isar, Technical University of Munich, Germany. The system concomitantly generates an attenuation and dark-field image. Since this is a prototype system, the potential for radiation dose optimization has not been thoroughly investigated yet. For all examinations involving ionizing radiation, the “As Low As Reasonably Achievable” (ALARA) principle demands the identification of the lowest radiation dose that still provides sufficient diagnostic image quality. Hence, radiation dose optimization represents an essential step in the responsible development of any new x-ray–based imaging modality.

Currently, acquiring a posteroanterior thorax image at this prototype system exposes a male reference person (73 kg, 173 cm) to an effective dose of 35 µSv [11]. However, a previous study [12] using simulated dose reduction reported that decreasing the radiation dose to 10.5 µSv for dark-field chest radiographs does not affect the diagnostic accuracy for the detection of COVID-19 pneumonia. Since the dose-reduction algorithm they used may produce stripe artifacts that would not occur in actual low-dose measurements, radiation doses even lower than 10.5 µSv might be feasible. To date, no other studies have investigated the potential of radiation dose reduction in dark-field chest radiography.

The present study investigates the impact of lower radiation doses on the diagnostic potential of dark-field radiographs for emphysema detection using the same simulation-based approach as described in [12]. In doing so, we aim to take a further step toward investigating the potential for dose optimization in dark-field chest radiography. Owing to its ability to visualize microstructural lung changes at the alveolar level, dark-field radiography has the potential to become a sensitive tool for early lung disease detection or triage, for example, in patients presenting with chest pain, cough, or dyspnea, or in ultra-low-dose lung assessment settings. In such contexts where examinations may be applied more broadly or potentially repeatedly, minimizing radiation exposure becomes substantially more relevant from both a clinical and regulatory perspective. Hence, establishing robust image acquisition even for reduced radiation doses represents an important methodological foundation for the continued clinical translation of the technique.

## Materials and methods

This study is a secondary analysis of a prospective monocentric study from our institution that investigates the influence of emphysema on the lungs’ dark-field signal. This underlying study was approved by the Institutional Review Board of the Technical University of Munich (Ethics Commission of the Medical Faculty, Technical University of Munich, Germany; 16/20 S) and the national radiation protection agency (Federal Office for Radiation Protection, Oberschleißheim, Germany, Z5-22462/2-2017-021) and has been conducted in accordance with the Declaration of Helsinki (as revised in 2013). All participants gave prior written informed consent for study participation in the underlying study and for any further analysis of their data.

### Underlying prospective COPD study

**Fig. 1:**
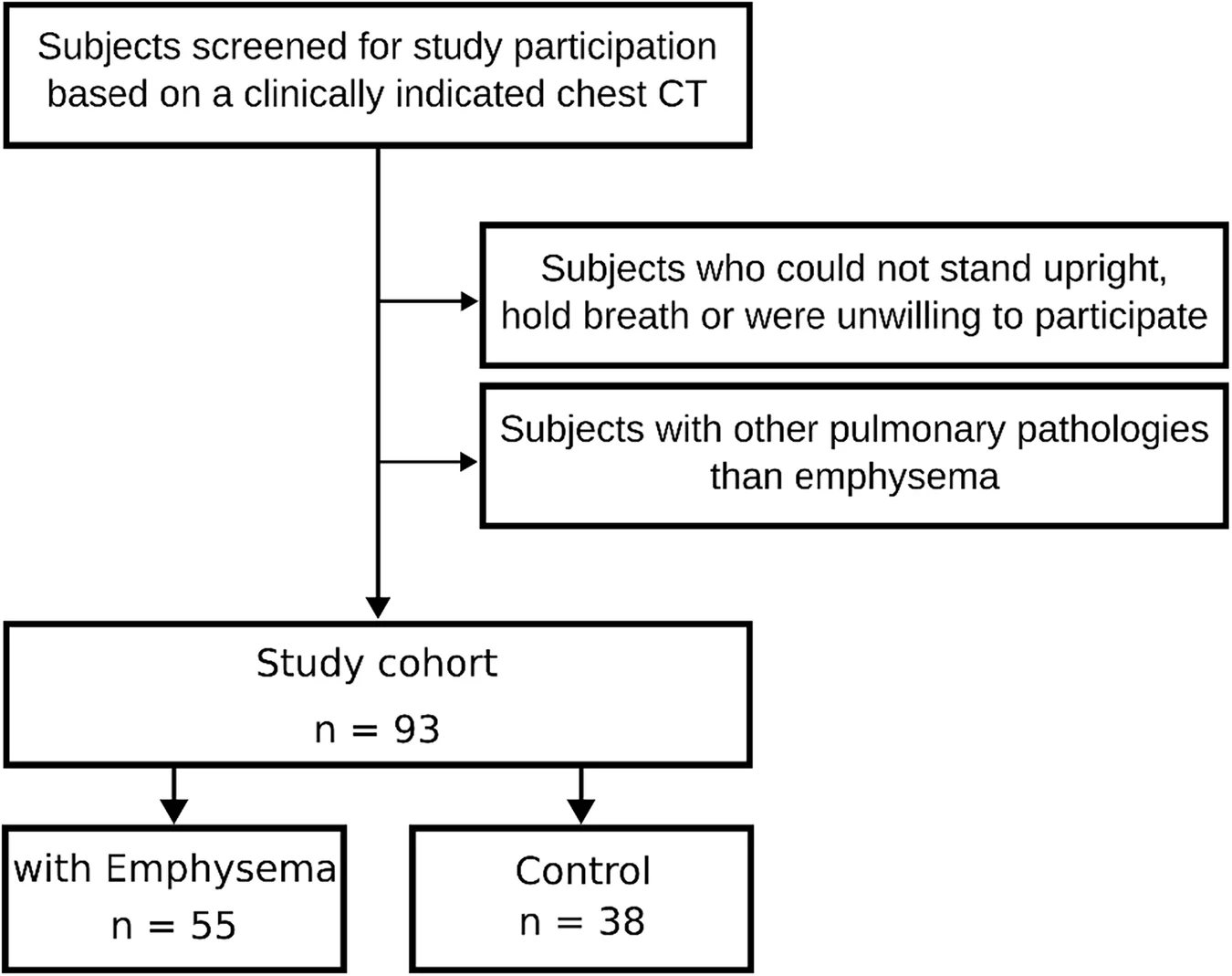
Flowchart for the selection of study participants

Between October 2018 and September 2023, clinically indicated computed tomography (CT) images of potential study participants were screened for study participation. Patients were eligible for study participation if their CT showed no changes in the lung tissue other than pulmonary emphysema (*i.e.*, no lung cancer, pleural effusion, atelectasis, air-space disease, ground-glass opacities, and pneumothorax). Patients without any changes in the lung tissue formed the control group. Further inclusion criteria were the ability to consent and to stand upright without assistance. For both the emphysema and the control group, exclusion criteria were being younger than 18 years and pregnancy (see Fig. 1).

CT imaging was performed with routine clinical protocols at one of three CT scanners (Philips iCT, Siemens SOMATOM, or Philips IQon Spectral CT). The CT images were reconstructed with a lung-specific convolution kernel and 3-mm slice thickness.

### Dark-field radiography

The prototype for x-ray dark-field scanning of the human thorax is located at the University Hospital Rechts der Isar, Technical University of Munich, Germany. During a 6.5-s scan, the system acquires a series of 195 raw x-ray images, from which both an attenuation and a dark-field radiograph are reconstructed. The prototype system uses a diagnostic x-ray tube operating at 70 kVp and a flat-panel detector with a field of view of 42 × 42 cm^2^ (see [1, 6, 13] for details). During image acquisition, participants stand and hold their breath at full inspiration. The median effective radiation doses of the participant cohort were 36 µSv (interquartile range: 26.1–45.6 µSv) for posteroanterior and 45 µSv (interquartile range: 33.0–57.7 µSv) for lateral projections, respectively.

### Simulated dose reduction

The simulated low-dose attenuation and dark-field radiographs were generated by a simulation algorithm that has been developed based on a motion artifact correction algorithm [14] and is described and evaluated in [15]. It has also already been employed in the evaluation of COVID-19 detection with simulated low-dose dark-field radiographs [12]. This low-dose algorithm virtually reduces the number of evaluated data points of the raw data, thereby mimicking the effect of a lower radiation dose per exposure.

### Reader study

As ground truth for each participant’s lung condition, radiologists independently assessed all CT images for emphysema severity according to the Fleischner scale (absent, trace, mild, moderate, confluent, advanced destructive) [16]. For 88 study participants, this was done by three radiologists (Jannis H. Bodden., A.P.S., and Alexander A. Fingerle). Here, the same dataset was used as in [10]. For five additional study participants, the radiologists were exchanged (A.W.M., F.T.G., and D.P.). For each participant, the median of the three ratings was considered the ground truth.

The measured and simulated attenuation and dark-field images were assessed in a reader study. For each participant, both image contrasts were displayed side by side with window settings optimized for lung visualization for both modalities.

Three radiologists (A.W.M., F.T.G., and D.P.) with 2, 7, and 16 years of experience performed a reader study. Based on both image contrasts, each radiologist grouped each study participant into one of the categories “absent or trace emphysema”, “mild emphysema”, “moderate emphysema”, and “severe emphysema”. Furthermore, each radiologist rated the image quality of the dark-field and attenuation radiograph as “0, not suitable for diagnostics (severe artifacts)”, “1, bad”, “2, fair”, “3, good”, or “4, excellent”. The reader study consisted of five evaluation rounds: In the first round, only the images corresponding to 20% of the radiation dose were evaluated. After a minimum break of 2 weeks, the 30% images were evaluated. Following the same pattern, the 40%, 50%, and the original, full-dose images were evaluated. The order of the images was randomized for each dose level. The readers were blinded to the participants’ personal and clinical data and to the dose level. The readers did not receive any feedback on their image evaluation between the different rounds.

### Quantitative and statistical analysis

For statistical tests, a *p*-value of less than 0.05 was considered statistically significant. For the analysis of the reader study, the answers of all three readers were pooled. Statistical analysis was performed by algorithms written in the programming languages Python, version 3.8.5 [17], and R, version 4.4.2 [18]. The area under the curve (AUC) of the receiver operating characteristic (ROC) was calculated using Obuchowski’s method for correlated and clustered data [19] to account for the statistical effects of our multi-reader-multi-case study design.

To test for significant differences between the assessment of image quality, the clustered Wilcoxon signed rank test implemented in the R library “clusrank” (version 1.0-4) was used [20].

Krippendorff’s alpha [21] was calculated to assess the inter-rater reliability of the emphysema classification.

The dark-field coefficient (DFC) was calculated for all images at each dose level by integrating the dark-field signal over each pixel of the lung area and dividing it by the lung volume estimated based on the attenuation radiographs as described in [22]. Hence, it is proportional to the number of traversed tissue-air interfaces [2]. By testing every numerical value of the dark-field coefficient in steps of 0.005 as a threshold value between healthy lungs and lungs with at least mild emphysema, the ROC curve for every dose level was determined. For each ROC curve, the maximum Youden’s index [23] was determined using the R package “cutpointr” (version 1.2.0). The maximum Youden’s index was used as a criterion for the optimum threshold of the dark-field coefficient to distinguish between controls and study participants with at least mild emphysema.

## Results

### Participant characteristics and simulated images

Dark-field intensity data of a total of 93 participants (57 male, 36 female) were included in this study, with a participants’ median age of 66 years (interquartile range 54 to 73 years) and a median body weight of 72 kg (interquartile range: 64–83 kg), see Table 1. Some of these study participants were also studied in previous publications: [1–3, 10] investigated the impact of emphysema on the dark-field signal using up to 88 study participants that were also included here. The participants used as controls in [6, 7, 12] were also included here.

**Table 1:** Participant characteristics

Emphysema severity	Number of participants (men/women)	Age, years	Weight, kg
All	93 (57/36)	66 (54–73)	72 (64–83)
Absent	38 (22/16)	63 (54–71)	79 (68–89)
Trace	22 (14/8)	66 (55–70)	72 (63–81)
Mild	11 (5/6)	70 (59–75)	67 (59–74)
Moderate	9 (6/3)	70 (55–71)	74 (65–86)
Confluent	9 (6/3)	73 (65–79)	63 (56–70)
Advanced destructive	4 (4/0)	64 (61–69)	66 (60–75)

Data are reported as medians (interquartile range). Emphysema severity was classified according to the grading system of the Fleischner Society [16]

**Fig. 2:**
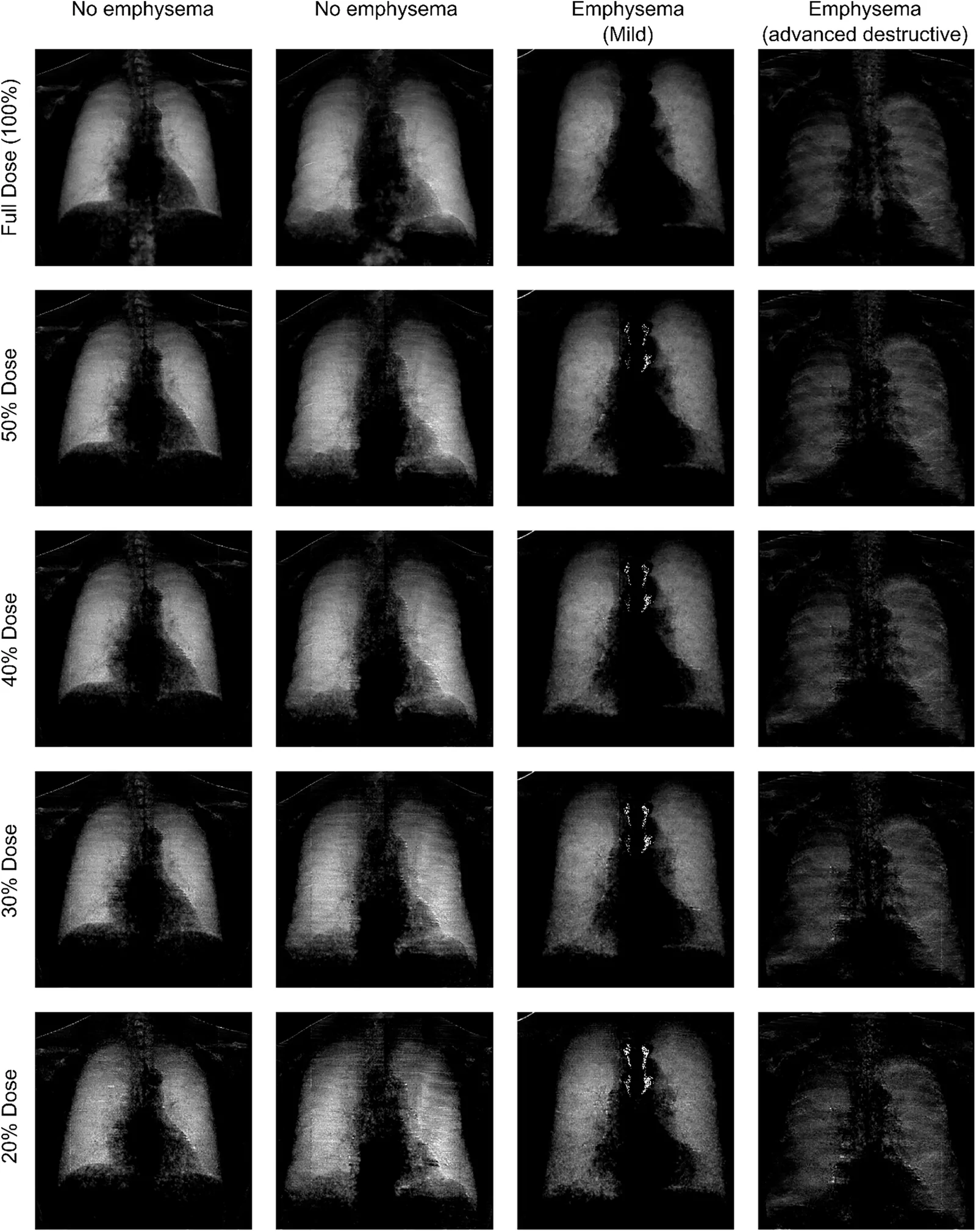
Dark-field images of four exemplary participants: two participants without lung disease and two participants with emphysema (mild and advanced destructive, respectively). The top row shows the images taken at the original, full dose of 100%, and the lower rows show the dark-field images with simulated dose levels of 50%, 40%, 30%, and 20%, respectively. All images are displayed with the same window levels

**Fig. 3:**
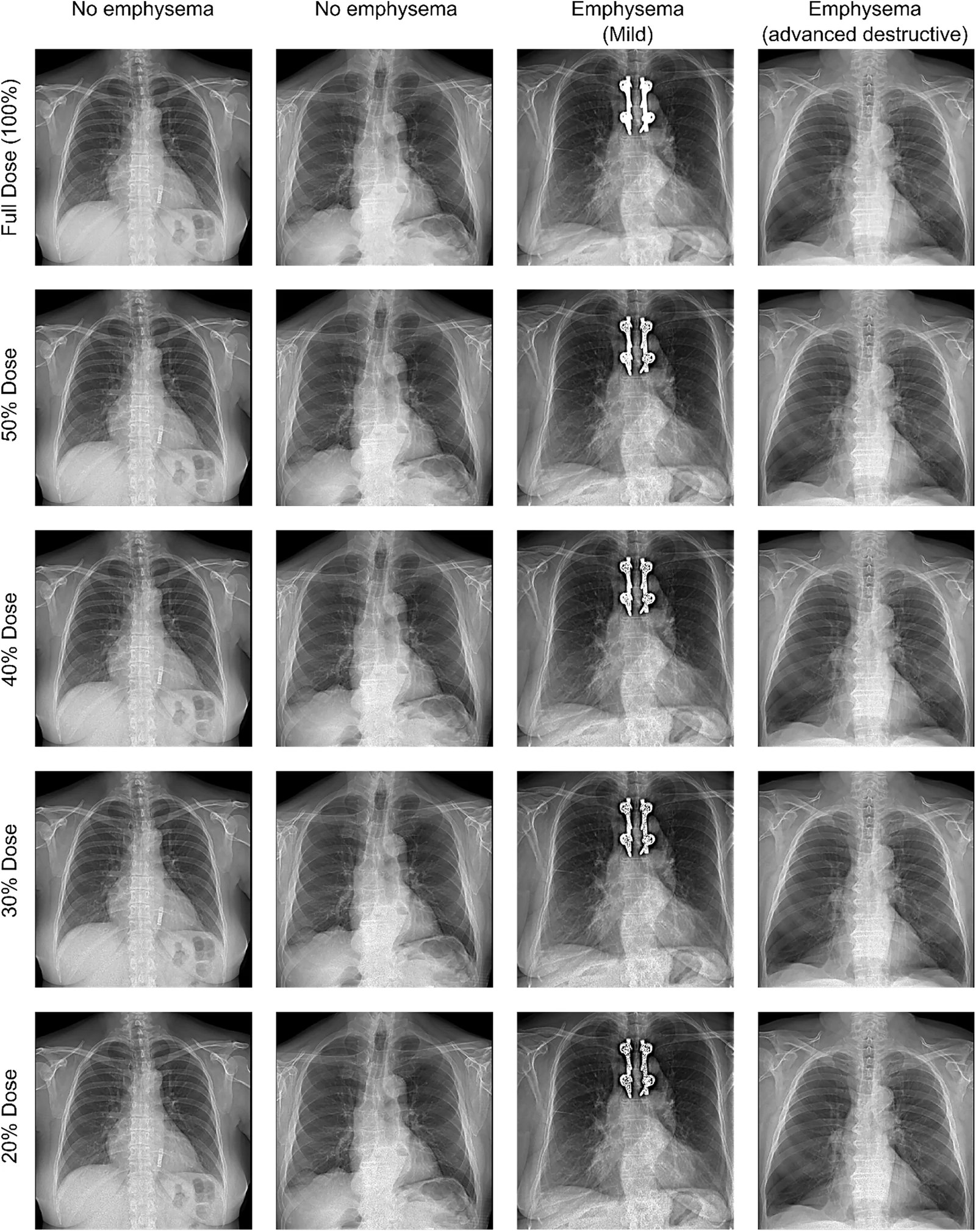
Attenuation images of four exemplary participants (same as in Fig. 2): two participants without lung disease and two participants with emphysema (mild and advanced destructive, respectively). The top row shows the images taken at the original, full dose of 100%, and the lower rows show the attenuation images with simulated dose levels of 50%, 40%, 30%, and 20%, respectively. All images are displayed with the same window levels

The simulated dark-field and attenuation images for reduced dose levels, along with the original, full-dose images, are shown in Figs. 2 and 3, respectively. For all dark-field images, the noise level increased with decreasing radiation dose. The 20% and 30% images show bright spots at the periphery of the lungs that are absent in the higher-dose images. Furthermore, horizontal stripe artifacts occurred with decreasing dose level for the dark-field images in the second column of Fig. 2. These stripe artifacts affect the perception of the homogeneity of the dark-field signal compared to the full-dose image. In the case of the third exemplary participant (third column), contrary to the 100% image, the corresponding simulated dark-field images show a false dark-field signal caused by foreign bodies in the spine.

For the attenuation images, dose reduction leads to an increased noise level visible in the abdominal area and behind strongly attenuating foreign bodies (see Fig. 3, third column). Despite that, no artifacts or other striking differences between full-dose and low-dose images can be seen.

### Reader study analysis

**Fig. 4:**
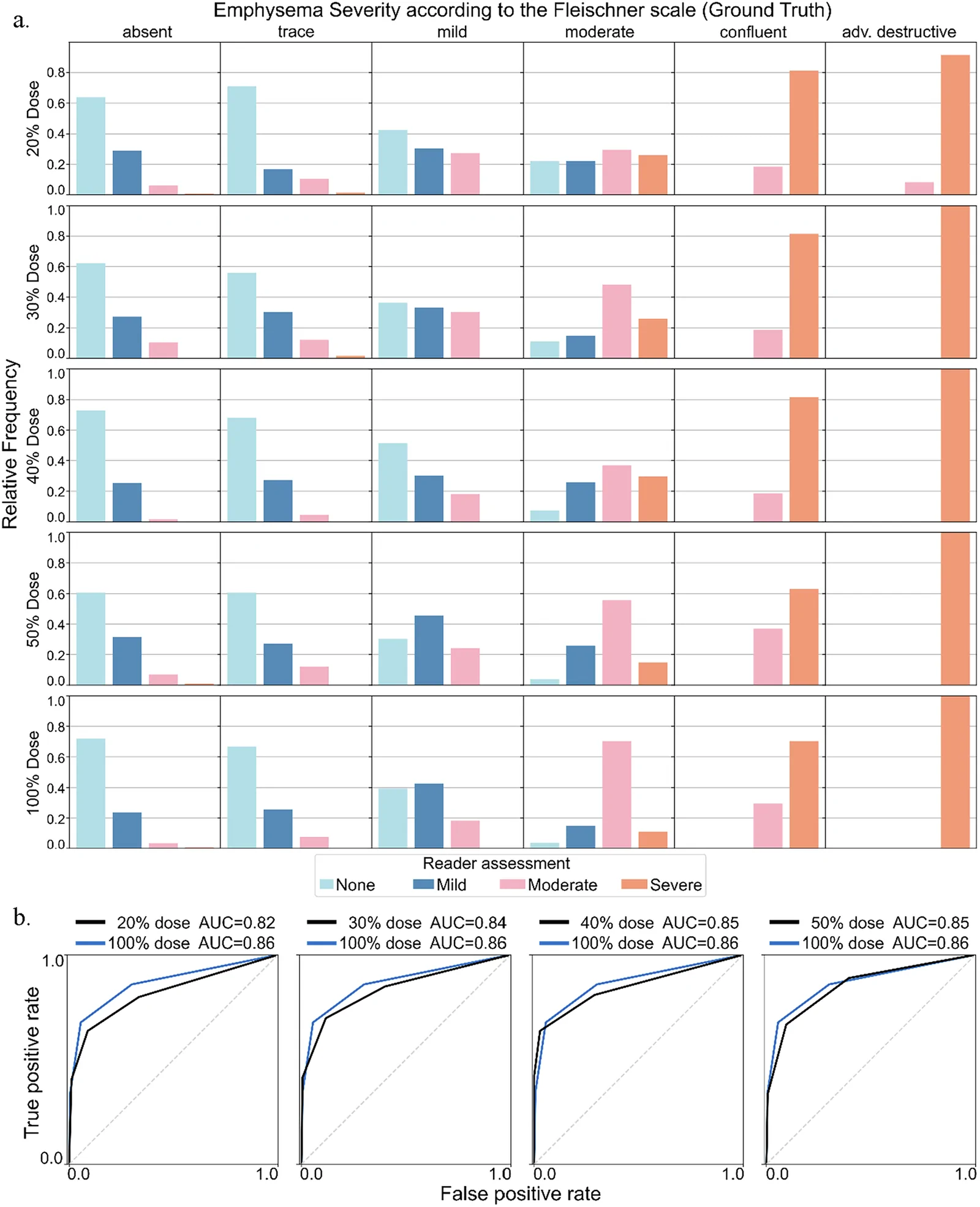
**a** Frequency distributions of reader ratings for color-coded emphysema severity (none, mild, moderate, and severe) for different stages of emphysema classified according to the Fleischner scale (absent, trace, mild, moderate, confluent, and advanced destructive). **b** Receiver operating characteristic (ROC) curves for the reader study, comparing the readers’ performance at each reduced dose level to the full-dose ROC curve. AUC, Area under the curve

The frequency of the readers’ ratings is shown in Fig. 4a. For the measured full-dose radiographs, the AUC of the ROC curve is 0.86 (95% CI: 0.80–0.93). For the reduced dose radiographs at 50%, 40%, and 30% of the full dose, respectively, no significant change in diagnostic accuracy could be observed (Fig. 4b). For radiographs at 20% of the full radiation dose, the AUC slightly decreased to 0.82 (95% CI: 0.74–0.90) (*p* = 0.048). The AUC values for all examined dose levels are shown in Table 2. Inter-reader reliability for emphysema classification was evaluated using Krippendorff’s alpha and ranged from 0.66 to 0.76 across the examined dose reductions (see Table 2).

**Table 2:** Reader study evaluation

Dose level	AUC	95% confidence interval	*p*-value	Krippendorff’s alpha
100%	0.86	(0.80, 0.93)	-	0.71
50%	0.85	(0.78, 0.92)	0.590	0.71
40%	0.85	(0.77, 0.93)	0.354	0.76
30%	0.84	(0.76, 0.91)	0.177	0.69
20%	0.82	(0.74, 0.90)	**0.048**	0.66

Area under the receiver operating characteristic curve (AUC) for different dose levels, the associated *p*-value for the difference between the simulated dose levels and the full dose, and Krippendorff’s alpha for each dose level. Significant *p*-values are marked in bold

**Fig. 5:**
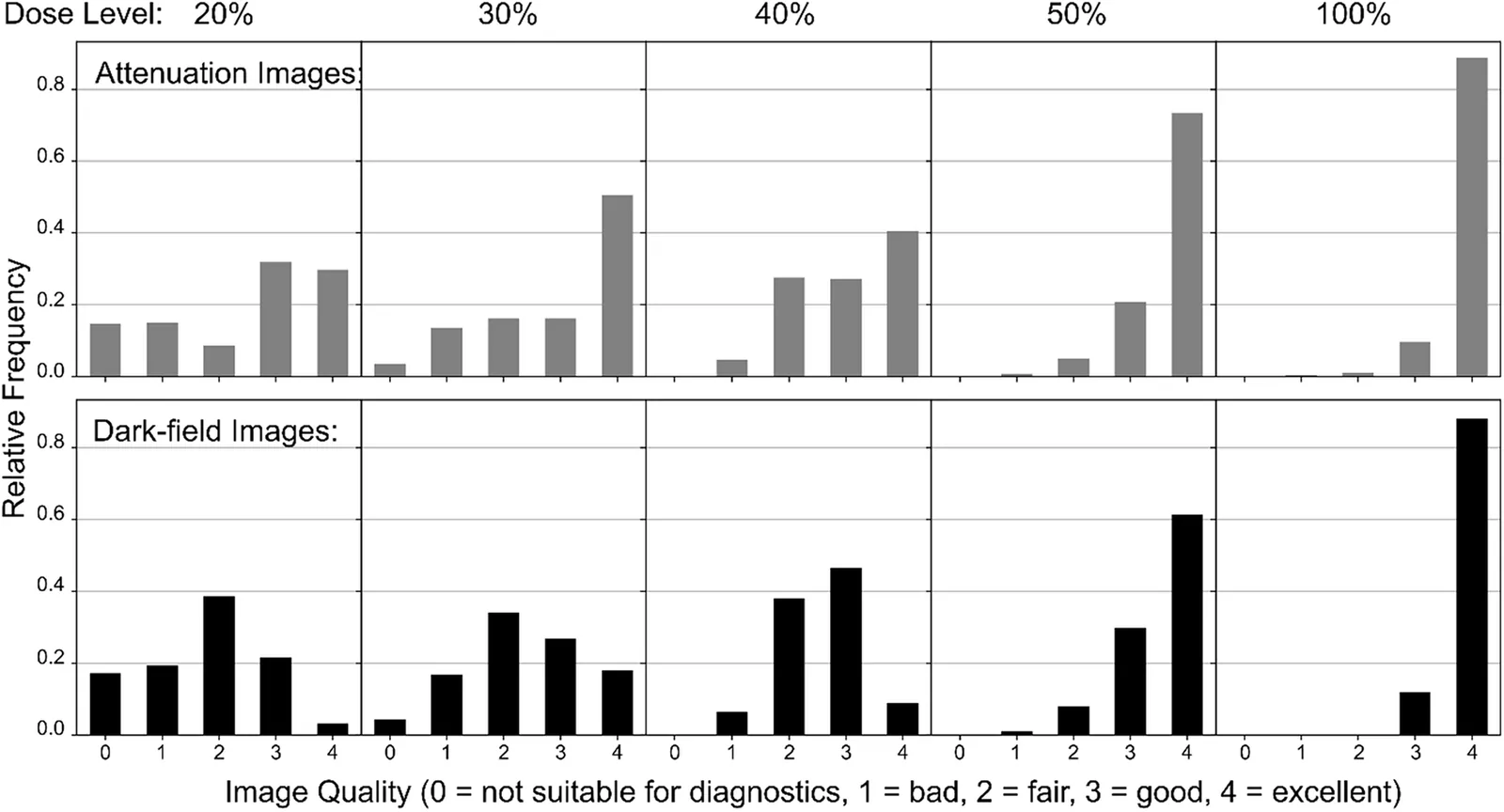
Reader-rated image quality for the dark-field and attenuation images of all dose levels examined

When assessing the measured full-dose attenuation and dark-field radiographs, the radiologists rated about 90% of the images as “excellent image quality” (see Fig. 5). With decreasing dose level, image quality was rated worse. For the 20% and 30% images, some attenuation and dark-field images were rated as not suitable for diagnostics due to severe artifacts. Statistically, the subjective assessment of image quality differed from the assessment at 100% for all simulated dose levels (*p* < 0.001 for all).

### Quantitative analysis

**Fig. 6:**
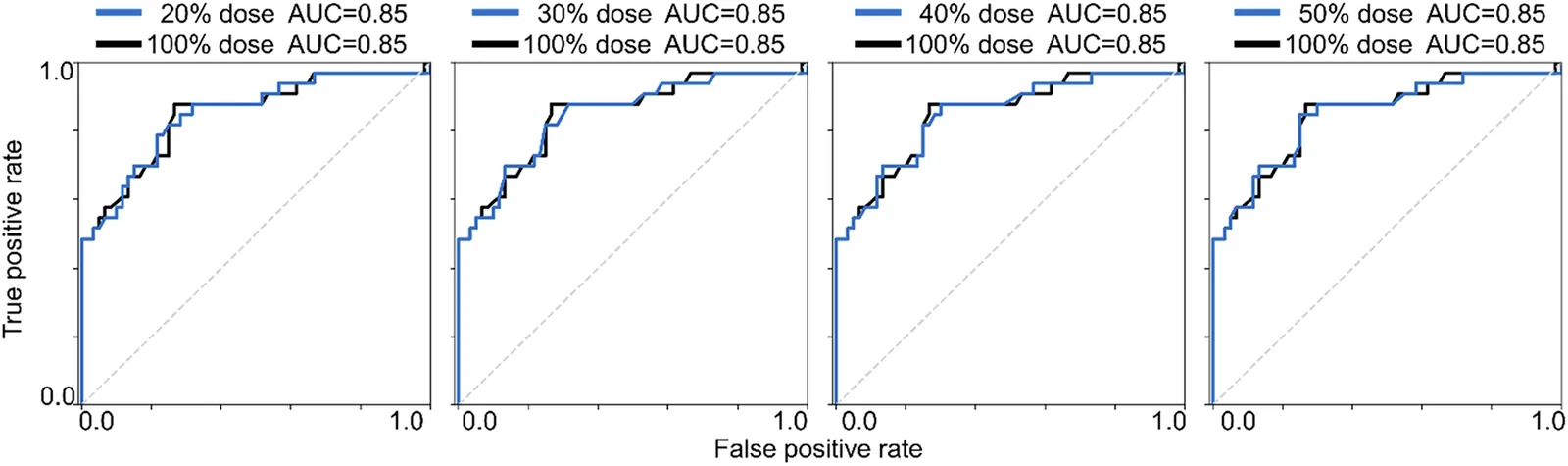
Resulting receiver operating characteristic curves when using the quantitative dark-field coefficient to differentiate between absent and at least mild emphysema for all examined dose levels. AUC, Area under the curve

For all dose levels, the AUC value of the ROC curve for detecting at least mild emphysema based on the dark-field coefficient was 0.85 (see Fig. 6). For the 100% images, the maximum Youden’s index was 0.61 with an optimal threshold of the dark-field coefficient of 2.23. The maximum Youden’s index changed only slightly for the simulated images, with a value of 0.60 for the 50% images (18 µSv) and 0.58 for 40% (14 µSv) to 20% (7 µSv). The corresponding optimal thresholds are 2.22 for 50%, 2.24 for 40%, 2.26 for 30%, and 2.21 for 20% of the original radiation dose of 36 µSv (see Table 3).

**Table 3:** Quantitative evaluation

Dose level	AUC	Maximum Youden’s index	Optimal threshold
100%	0.85	0.61	2.23
50%	0.85	0.60	2.22
40%	0.85	0.58	2.24
30%	0.85	0.58	2.26
20%	0.85	0.58	2.21

Analysis of the area under the receiver operating characteristic curve (AUC) when using different thresholds of the dark-field coefficient to distinguish between absent and at least mild emphysema

## Discussion

In this study, we investigated how reduced radiation doses would affect the performance of radiologists to detect emphysema using dark-field radiography. For this purpose, a reader study with measured full-dose and simulated dose-reduced attenuation and dark-field images was conducted. When calculating the area under the curve of the radiologists’ ROC curves, which is a measure of the radiologists’ performance for emphysema detection and classification, there was no significant difference between images acquired with a median effective dose of 36 µSv (interquartile range: 26.1–45.6 µSv) and simulated low-dose images corresponding to 30% of these full-dose images, *i.e*., low-dose images with a median effective dose of 11 µSv (interquartile range: 7.8–13.7 µSv). With simulated images corresponding to a median effective dose of 7 µSv, *i.e*., 20% of the original dark-field radiographs, radiologists yielded a decreased AUC of 0.82 compared to the full-dose images (*p* = 0.048). While an AUC of 0.82 can still be regarded as an excellent diagnostic performance, our objective is to reduce the radiation dose without affecting diagnostic performance. Additional quantitative analysis using the so-called dark-field coefficient, proposed by [2] to distinguish between absent and at least mild emphysema, yielded an AUC = 0.85 for all dose levels.

For comparison, chest CTs typically cause effective doses of 5–14 mSv and literature values for conventional posteroanterior chest radiography range from 10 to 100 µSv, with values differing between countries [24–27]. Consequently, the radiation associated with our simulated dark-field images corresponds to the lower bound of conventional radiography, while still enabling excellent diagnostic performance (AUC = 0.84) in the assessment of emphysema. Although the currently used effective dose of 40 µSv for dark-field radiography falls within the range of reported radiation doses for conventional radiography, no diagnostic reference values have yet been established for dark-field imaging. Hence, exploring the potential for radiation dose reduction is an important step toward fulfilling the ALARA principle. However, Krippendorff’s alpha being between 0.67 and 0.76 suggests only moderate reliability of our findings, indicating non-negligible variability in emphysema classification. Although the small differences in AUC values between full-dose images and the 50% to 30% dose images were not statistically significant (*p* ≥ 0.177), a large inter-reader variability could mask true significant differences due to dose reduction. Given our modest cohort size, the absence of significant differences in AUC values in combination with a large inter-reader variability requires further investigations. A larger cohort would strengthen our results by increasing statistical power and by enabling a study design using different subsets of participants for different dose levels to eliminate the risk of bias due to the reader memorizing and recognizing the patient’s chest radiographs. Here, a minimum period of 2 weeks between reading sessions and randomizing the order of the images in each session was used to reduce this risk, but a different study design would be favorable.

The observed decrease in diagnostic performance can mainly be attributed to a reduced image quality due to simulation-induced image artifacts. As reported by [12], the employed algorithm for dose reduction caused image artifacts in some dark-field images, which was also reflected in the subjective assessment of image quality. However, Bast et al [15] showed that horizontal stripe artifacts are solely due to the employed simulation algorithm. Corresponding real low-dose measurements of an anthropomorphic chest phantom did not exhibit such artifacts. Hence, we expect that real low-dose acquisitions of study participants would display an increased noise level but no stripe artifacts, which might enable even lower radiation doses than 11 µSv without significant loss of diagnostic accuracy. Consequently, our findings provide only a lower bound on achievable dose reduction. While the algorithm reduces the number of x-ray exposures per pixel, real dose reduction would be implemented by reducing the x-ray tube current. Although both approaches mathematically yield the same total radiation dose, they impact the image reconstruction differently [15]. As the algorithm-induced artifacts are a major limitation of our findings, a study with real low-dose patient images is strictly necessary.

Furthermore, the generalizability of our findings to real-world clinical populations might be reduced, since our study neither included participants with lung diseases other than emphysema nor with comorbidities existing besides emphysema. Our relatively homogeneous cohort was intentionally chosen to evaluate the methodological reliability of ultra-low-dose dark-field imaging specifically for emphysema. Emphysema, with its varying degrees of microstructural destruction of lung tissue, produces a broad range of dark-field signal intensities. This makes this pathology particularly suitable for assessing the robustness of the method across the relevant signal spectrum in a controlled setting. Future studies need to evaluate dark-field imaging for more heterogeneous patient populations. Another interesting aspect for future work might be combining dark-field imaging and quantitative CT metrics. In our study, we used the Fleischner visual grading system [16] as a reference standard, as a CT-based emphysema index is known to have limited sensitivity for mild and moderate emphysema [1].

In conclusion, the radiologists’ ability to diagnose and stage emphysema using simulated dark-field radiographs corresponding to 11 µSv suggests that reducing the radiation dose of dark-field chest radiography is feasible. Our preliminary results can serve as the foundation for further studies with low-dose imaging of patients. While real low-dose studies with larger cohorts are needed to confirm our findings, our results are an important step toward determining the radiation dose that is as low as reasonably achievable for dark-field chest radiography by providing an estimate of which levels are relevant for further investigation. While dark-field radiography is not intended to replace CT, its combination of very low radiation dose and sensitivity to lung microstructure may create interesting perspectives for future applications. For example, ultra-low-dose dark-field imaging could potentially support early detection or longitudinal monitoring of microstructural lung alterations in selected patient groups, where repeated examinations and minimal radiation exposure are desirable.

## Data Availability

Data generated or analyzed during the study are available from the corresponding author upon request.

## Abbreviations

AUC: Area under the ROC curve
ROC: Receiver operating characteristic
